# Monoclonal antibody exposure in rat and cynomolgus monkey cerebrospinal fluid following systemic administration

**DOI:** 10.1186/s12987-018-0093-6

**Published:** 2018-03-20

**Authors:** Qin Wang, Luisette Delva, Paul H. Weinreb, Robert B. Pepinsky, Danielle Graham, Elvana Veizaj, Anne E. Cheung, Weiping Chen, Ivan Nestorov, Ellen Rohde, Robin Caputo, Geoffrey M. Kuesters, Tonika Bohnert, Liang-Shang Gan

**Affiliations:** 1 Biogen, Inc., 250 Binney Street, Cambridge, MA 02142 USA; 2 Present Address: Ribon Therapeutic, 99 Hayden Ave #100, Lexington, MA 02421 USA; 3 Present Address: Intellia Therapeutics, 40 Erie St, Cambridge, MA 02139 USA; 4 Present Address: Thermo Fisher Scientific Inc, 790 Memorial Dr, Cambridge, MA 02139 USA; 5 Present Address: Merrimack pharmaceuticals, 1 Kendall Square, Cambridge, MA 02139 USA; 6 Present Address: Foresee pharmaceuticals, 1 Innovation Way, Suite 100, Newark, DE 19711 USA

## Abstract

**Background:**

Many studies have focused on the challenges of small molecule uptake across the blood–brain barrier, whereas few in-depth studies have assessed the challenges with the uptake of antibodies into the central nervous system (CNS). In drug development, cerebrospinal fluid (CSF) sampling is routinely used as a surrogate for assessing CNS drug exposure and biomarker levels. In this report, we have studied the kinetic correlation between CSF and serum drug concentration–time profiles for five humanized monoclonal antibodies in rats and cynomolgus monkeys and analyzed factors that affect their CSF exposure.

**Results:**

Upon intravenous (IV) bolus injection, antibodies entered the CNS slowly and reached maximum CSF concentration (^*CSF*^*T*_*max*_) in one to several days in both rats and monkeys. Antibody serum and CSF concentration–time curves converged until they became parallel after ^*CSF*^*T*_*max*_ was reached. Antibody half-lives in CSF (^*CSF*^*t*_*½*_) approximated their serum half-lives (^*serum*^*t*_*½*_). Although the intended targets of these antibodies were different, the steady-state CSF to serum concentration ratios were similar at 0.1–0.2% in both species. Independent of antibody target and serum concentration, CSF-to-serum concentration ratios for individual monkeys ranged by up to tenfold from 0.03 to 0.3%.

**Conclusion:**

Upon systemic administration, average antibodies CSF-to-serum concentration ratios in rats and monkeys were 0.1–0.2%. The ^*CSF*^*t*_*½*_ of the antibodies was largely determined by their long systemic *t*_*½*_ (^*systemic*^*t*_*½*_).

## Introduction

The blood brain barrier (BBB) is the single most significant obstacle that impedes the delivery of therapeutics to targets in the central nervous system (CNS). For medications that interact with targets in the CNS, it is essential for therapeutic and/or safety reasons to obtain accurate kinetic relationships between CNS and systemic circulation levels. It is also important to understand the factors that affect this relationship in order to project CNS drug levels based on their systemic concentration–time profiles. In practice, cerebrospinal fluid (CSF) is the most accessible and widely used sampling matrix for measuring drug and biomarker levels in the CNS. CSF sampling is inconvenient, and therefore requires rigorous study design to minimize the number of time points that need to be collected. Several excellent reviews highlight the scientific rationale and utility of using CSF as a surrogate for assessing CNS drug exposure levels [1–5]. Although there are numerous studies examining the rates of small molecule drug penetration across the blood brain barrier (BBB) and blood CSF barrier (BCSFB), there are few in-depth studies looking at the kinetics of antibody transmission into and out of CSF, especially with respect to the key factors that govern CSF antibody exposure. Considerations include the time needed for an antibody to reach maximum CSF drug concentration (^*CSF*^*T*_*max*_), antibody CSF half-life (^*CSF*^*t*_*½*_) and the length of time required for achieving steady-state antibody levels between CSF and systemic circulation. In this study, we report the kinetic relationships of CSF versus serum concentration–time profiles of five humanized monoclonal antibodies in rats and cynomolgus monkeys following systemic administration and factors that affect their CSF antibody exposures. These findings have important implications for assessing CNS antibody drug kinetics in humans.

## Materials and methods

### Materials

In this study, we tested five human monoclonal antibodies (mAb). All five antibodies are highly selective and have subnanomolar affinities for their respective targets. Antibody A (BIIB054) preferentially binds to aggregated forms of α-synuclein and binds human, non-human primate (NHP), and rat α-synuclein with similar affinity. Antibody B (399 H0/L0) recognizes JC virus, which is found in humans [6], but is not present in NHP or rats. Antibody C (BIIB076) and antibody D (40E8) bind to the microtubule-binding protein tau. Antibody C recognizes a linear sequence within tau and binds to human and NHP, but not rat tau. Antibody D is specific for phosphorylated tau and recognizes phosphorylated human, NHP, and rat tau. Antibody E (BIIB033, opicinumab) binds LRR- and Ig-domain-containing Nogo receptor interacting protein 1 (LINGO-1) and exhibits similar affinity for human, NHP, and rat LINGO-1. Antibodies A, C, D, and E are fully human antibodies, and antibody B is a humanized rabbit antibody. Antibodies, A, B, C, and D contain a wildtype human IgG1 Fc, while antibody E contains an aglycosylated human IgG1-Fc designed to reduce effector function [7]. Antibody A (BIIB054), antibody C (BIIB076), and antibody E (BIIB033) are currently being assessed in clinical trials.

### Methods

Rat PK studies. Adult male Sprague–Dawley rats (250–300 g) (Charles-River Laboratory, Wilmington, MA) were housed at constant temperature (22 ± 2 °C) and relative humidity (50–70%) under a regular light/dark schedule (light, 7:00 A.M. to 7:00 P.M.). Food and water were freely available. The dose administration route was either by tail vein (mAbs A, B, C and D) or intraperitoneal injection (mAb E). At various time points after dose administration, the animals were euthanized by CO_2_ asphyxiation and immediately followed by CSF collection via cisterna magna puncture and blood collection via cardiac puncture. The rats used to evaluate antibody E were adult male Brown Norway rats (average weight 150–200 g). The procedures were essentially the same. For brain and spinal cord collection, perfusion was performed promptly following CSF/blood collection with 15 mL of saline via a peristaltic pump inserted into the left ventricle. Whole blood samples were left on the bench for about 15–30 min and spun at 3000 rpm (approximately 1000×*g*) for 10 min to isolate serum. All samples were stored at − 80 °C until analysis. All experimental procedures were approved and monitored by Biogen’s Institutional Animal Care and Use Committee (IACUC).

Studies in NHP were carried out at SNBL (Everett, WA) with approved IACUC accreditation. Briefly, young adult cynomolgus monkeys (2.5–4 kg), were individually housed in cages that complied with the Animal Welfare Act, corresponding guidelines and standard operating procedures (SOPs) of SNBL. Approximately 1 week prior to dose administration, a single lumbar intrathecal catheter (advanced cranially into the thoracic region) was surgically implanted to allow repeated CSF sampling. Prior to dose administration, CSF was collected from the catheter for baseline measurements. The intravenous (IV) dose was administered via primed butterfly infusion lines over 1–2 min. At pre-selected sampling time points, blood (1 mL) and CSF (0.5 mL) were collected from restrained, conscious animals. Serum and CSF samples were prepared as described above for rat studies. CSF samples were visually examined for blood contamination and contaminated samples were excluded.

The concentrations of antibodies A–E in serum and CNS specimens were measured using enzyme-linked immunosorbent assays (ELISAs). A generic sandwich ELISA format similar to the one described [8] was optimized for testing of each antibody. Because the capture and detection reagents are human IgG specific rather than target specific, the assays could be used to quantify human or humanized IgG molecules in non-human biological matrices including NHP and rat samples with minor modifications [8, 9]. The assays measured total mAb concentrations that included both unbound and soluble ligand-bound antibody. Capture and detection reagents were purchased from Southern Biotech (http://www.southernbiotech.com). Briefly, for the analysis of antibody A–D samples, 96-well ELISA plates were coated with the capture antibody, monkey-adsorbed goat anti-human IgG, at 1 µg/mL. The plates were blocked and washed, and standards (STDs), quality controls (QCs), or study samples containing test articles were added to the plate. The plates were again washed, and a 1:7500 dilution (50 ng/mL) of enzyme conjugated detection antibody (goat anti-human IgG-HRP), was added to the wells. After washing, the HRP substrate tetramethylbenzidine (TMB) was added. The HRP reaction was stopped following the addition of 1 N H_2_SO_4_ and the absorbance at 450 nm was measured on a microplate reader. Standard calibration curves were generated and the concentrations of the controls and samples were interpolated from their respective standard curves. On average, the quantitation ranges of the ELISAs were between 16 and 4400 ng/mL. Lower limits of quantitation were generally 16 ng/mL for CSF and 50 ng/mL for serum samples. Antibody E samples were quantified as previously described [10]. Pharmacokinetic modeling was carried out using Phoenix 6.4.0 software (http://www.certara.com).

## Results

**Fig. 1:**
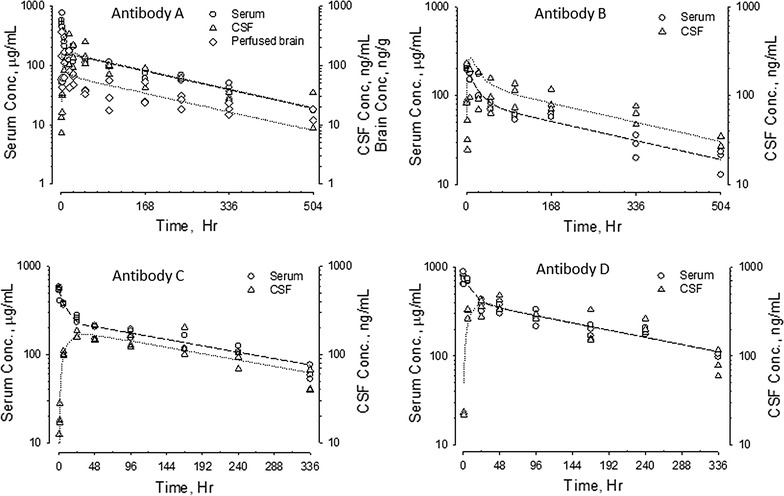
Serum and CSF concentration–time profiles of antibodies A (10 mg/kg), B (10 mg/kg), C (20 mg/kg) and D (20 mg/kg) in adult Sprague–Dawley rats following single dose IV administration. For antibody A, perfused brain concentrations were also determined. Left *y*-*axes* denote antibody serum concentration in μg/mL (open circle). Right *y*-*axes* denote antibody CSF or brain concentrations in ng/mL (open triangle). Data for each individual animal are plotted separately

**Fig. 2:**
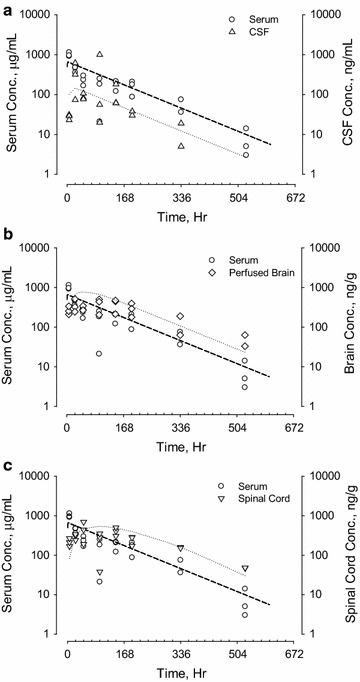
Serum and CNS tissue concentration–time profiles of antibody E in Brown Norway rats after a single intraperitoneal (IP) administration. Left *y*-*axes* denote serum concentration of antibody E in μg/mL (open circle). Right *y*-*axes* denote tissue concentrations of antibody E in ng/mL for CSF (open triangle, **a**) or ng/g of wet tissues for brain (open diamond, **b**) and spinal cord (open inverted triangle, **c**). Data for each individual animal are plotted separately

The pharmacokinetic properties of five human antibodies in serum and CSF were evaluated in rats and NHPs. A two-compartment first order pharmacokinetic model fit the concentration–time curves for all five antibodies well. Serum and CSF concentration–time profiles for antibodies A (10 mg/kg), B (10 mg/kg), C (20 mg/kg) and D (20 mg/kg) in rats following IV administration are shown in Fig. 1. Serum concentration profiles showed a multi-exponential decrease. All 4 antibodies appeared in the CSF slowly, with ^*CSF*^*T*_*max*_ ranging between 6 and 48 h. Serum and CSF concentration–time curves became parallel after ^*CSF*^*T*_*max*_. CSF antibody concentrations were approximately 1000-fold lower than serum concentrations. Since antibody E was dosed by intraperitoneal (IP) injection in Brown-Norway rats, the serum and CNS tissue concentration–time profiles for CSF, brain, and spinal cord are plotted separately in Fig. 2. Following IP administration (30 mg/kg), ^*serum*^*T*_*max*_ was observed 6–24 h after dosing. The ^*CSF*^*T*_*max*_, ^*brain*^*T*_*max*_ and ^*Spinal_ cord*^*T*_*max*_ were achieved 1–2 days following ^*serum*^*T*_*max*_. Like the data shown in Fig. 1 for IV dosing, serum and CSF concentration–time curves following IP administration also became parallel after ^*CSF*^*T*_*max*_. Concentration–time curves in brain (for antibodies A and E) and spinal cord (for antibody E) paralleled those in CSF, although the antibody concentrations in these CNS tissues were slightly higher than in CSF (Figs. 1, 2). No evidence for target-mediated clearance was observed, which is consistent with either the lack of cross-reactivity of the antibodies to rodent targets (antibodies B and C) or the low level of target in the periphery (antibodies A, D, and E).

**Fig. 3:**
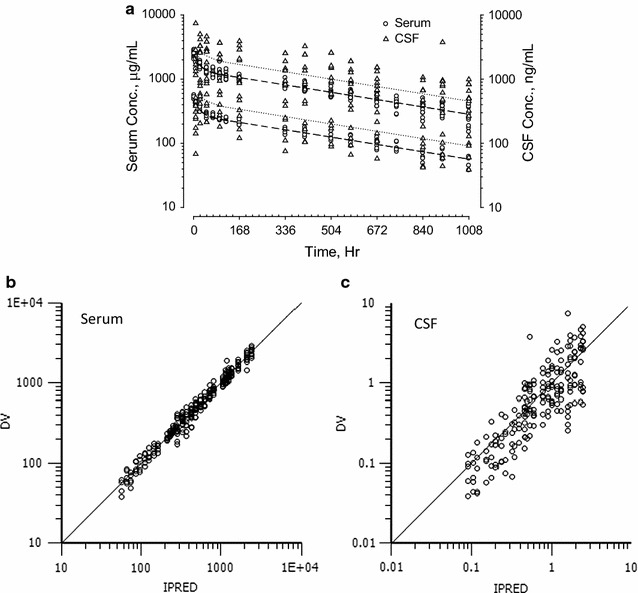
Serum and CSF concentration–time profiles of antibody A in adult male cynomolgus monkeys (**a**) after a single IV administration of 20 mg/kg (N = 4) or 100 mg/kg (N = 11). Left *y*-*axes* denote serum concentrations in μg/mL (open circle). Right *y*-*axes* denote CSF concentrations in ng/mL (open triangle). Data for each individual animal are plotted separately. Plots for observed (*y*-*axis*) versus predicted (*x*-*axis*) antibody A concentrations in serum (**b**) and CSF (**c**) are on log scales

**Fig. 4:**
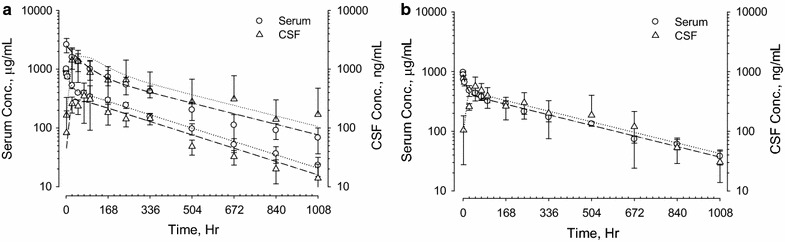
Mean serum and CSF concentration–time profiles in adult male cynomolgus monkeys following single IV dose administration of antibody C (**a**) at 30 mg/kg (N = 6) or 100 mg/kg (N = 12) and following a single IV administration of antibody D (**b**) at 30 mg/kg. Left *y*-*axes* denote serum concentrations in μg/mL (open circle). Right *y*-*axes* denote CSF concentration in ng/mL (open triangle)

**Fig. 5:**
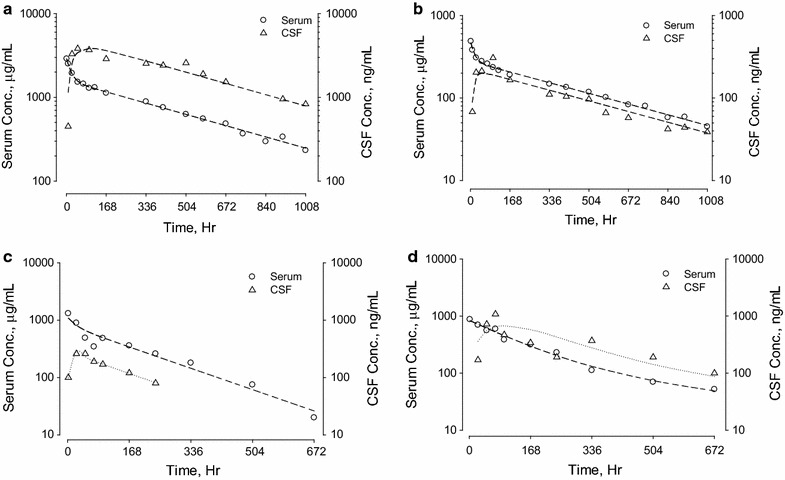
Representative serum and CSF concentration–time profiles of antibodies A and C from individual cynomolgus monkeys. **a** Represents antibody A at 100 mg/kg, **b** represents antibody A at 20 mg/kg, and **c**, **d** represent antibody C at 30 mg/kg in selective animals. Left *y*-*axes* denote serum concentrations in μg/mL (open circle). Right *y*-*axes* denote CSF concentration in ng/mL (open triangle)

Antibodies A, C and D were also evaluated in cynomolgus monkeys. The serum and CSF concentration–time profiles are plotted in Figs. 3, 4 and 5. As observed in rats, serum concentration profiles for each antibody in NHP showed a multi-exponential decrease. The antibodies appeared in the CSF gradually, peaking at 24–96 h after IV administration. Serum and CSF concentration–time curves became parallel after ^*CSF*^*T*_*max*_, and CSF antibody concentrations on average were approximately 1000-fold lower than serum concentrations. Figure 3 shows antibody A serum and CSF concentration time curves after single IV doses of 20 and 100 mg/kg. The serum concentrations were proportional to antibody dose and the CSF concentrations were proportional to the antibody serum concentrations. Figure 4 shows serum and CSF concentration time curves following IV dosing of antibody C at 30 and 100 mg/kg (panel a) and antibody D at 30 mg/kg (panel b). The pharmacokinetic profiles of antibodies A, C and D were similar. Although the inter-subject variations in serum concentration–time profiles for all three antibodies were relatively small, there was an approximately tenfold variation in CSF-to-serum concentration ratios. To better understand the variability seen in the CSF measurements, we analyzed serum and CSF concentration–time curves of individual animals from the three NHP studies. Representative results from four animals are shown in Fig. 5. For the individual animals, ^*CSF*^*t*_*½*_ and ^*serum*^*t*_*½*_ were similar, but the CSF-to-serum concentration ratios varied from 0.03 to 0.32%. The variation in CSF-to-serum ratios was random and occurred in all studies irrespective of intended target and dose strength.

Results of non-compartmental analysis (NCA) of the pharmacokinetic parameters for the five antibodies characterized in Figs. 1, 2, 3 and 4 are summarized in Table 1. All five antibodies exhibited high serum exposures (^s*erum*^*AUC*), and long half-lives (*t*_*½*_) in both serum and CNS tissues. Following IV administration, antibodies appeared in the CSF gradually, reaching maximum CSF concentration (^*CSF*^*C*_*max*_) between 16 and 48 h in rats and 2–3 days in cynomolgus monkeys (^*CSF*^*T*_*max*_). For all five antibodies, serum and CSF concentration–time curves became parallel 2–3 days after dose administration, with CSF concentrations roughly one thousand times lower than the corresponding serum concentrations and independent of dose. For example, antibody A had a ^*CSF*^*AUC*-*to*-^*serum*^*AUC* ratio of 0.15 ± 0.07% at 20 mg/kg (N = 4) and 0.16 ± 0.08% at 100 mg/kg (N = 11). Antibody C had an average ^*CSF*^*AUC*-*to*-^*serum*^*AUC* ratio of 0.09 ± 0.06% at 30 mg/kg (N = 12) and 0.10 ± 0.05% at 100 mg/kg (N = 6). Taking into consideration low and high dose groups, antibody A had relatively long ^*serum*^*t*_*½*_ of 18 ± 3 days and ^*CSF*^*t*_*½*_ of 21 ± 6 days in monkeys (N = 15). On the other hand, antibody C had a relatively short ^*serum*^*t*_*½*_ of 6 ± 2 days and ^*CSF*^*t*_*½*_ of 8 ± 3 days in monkeys (N = 18).

**Table 1:** Pharmacokinetic parameters of antibodies A, B, C, D and E in rodents and non-human primates

Test article	Animal info	Dose	Serum PK parameters^a^				CSF PK parameters^a^				^CSF^AUC/^Serum^AUC ratio (%)
			^serum^C_max_^c^	^serum^T_max_^c^	^serum^AUC_INF_	^serum^t_½_	^CSF^C_max_^c^	^CSF^T_max_^c^	^CSF^AUC_INF_	^CSF^t_½_	
		mg/kg	μg/mL	H	μg*h/mL	H	ng/mL	H	ng*h/mL	H	
A	Rat^b^	10 (IV)	560		40,400	155	200	16	38,000	185	0.09
B	Rat^b^	10 (IV)	208		31,700	234	110	24	48,400	245	0.15
C	Rat^b^	20 (IV)	778		108,500	169	380	48	94,000	136	0.09
D	Rat^b^	20 (IV)	559		67,100	173	170	24	48,000	143	0.07
E	Rat^b,d^	30 (IP)	1000	6	71,000	80	340	24	54,000	44	0.075
A	NHP (N = 4)	20 (IV)	520 ± 35		171,400 ± 23,100	402 ± 39	590 ± 270	42 ± 36	258,000 ± 124,600	423 ± 44	0.15 ± 0.07
	NHP (N = 11)	100 (IV)	2270 ± 650		854,700 ± 266,500	456 ± 87	2670 ± 2060	43 ± 30	1,441,200 ± 798,100	531 ± 152	0.16 ± 0.08
C	NHP (N = 11)	30 (IV)	1220 ± 390		163,200 ± 36,000	156 ± 34	680 ± 710	48 ± 29	140,300 ± 73,300	201 ± 71	0.09 ± 0.06
	NHP (N = 6)	100 (IV)	4210 ± 790		467,300 ± 76,500	126 ± 31	2210 ± 810	64 ± 29	538,300 ± 231,300	181 ± 39	0.10 ± 0.05
D	NHP (N = 4)	30 (IV)	980 ± 70		179,700 ± 22,400	290 ± 35	580 ± 240	60 ± 24	194,400 ± 106,800	257 ± 44	0.10 ± 0.04

^a^Pharmacokinetic calculation was based on linear-log noncompartmental analysis for both CSF and serum profiles

^b^ Rat PK parameters were based on mean data

^c^ Maximum serum and CSF levels were based on the highest observed serum and CSF concentration, respectively

^d^ Pharmacokinetic parameters from Ref. [7]

Upon entering the CNS, there are four possible clearance mechanisms: (1) movement back across the BBB and/or BCSFB via a first order non-specific process; (2) active/facilitated transport out of the CNS by efflux transporters located at the membrane sites of the BBB and/or BCSFB, an energy-requiring process against the antibody concentration gradient; (3) biotransformation/metabolism, and (4) convection carried by brain ISF/CSF flow and drainage. For mass balance at steady-state, solute concentration in the CNS is determined by the rate entering the CNS divided by the sum of the four possible clearance mechanisms out of the same compartment. To simplify the analysis, we treated the brain tissue and CSF as one fast equilibrium homogeneous compartment as shown in Eq. 1: where *R*_*in*_ is the rate of passage into the CNS in amount per time; *CL*_*out*_ is the total clearance out of the CNS in volume per time; ^*CSF*^*C* and ^*serum*^*C* denote the steady-state drug concentrations in CSF and systemic circulation, respectively; *PS*_*A to B*_ and *PS*_*B to A*_ represent directional first order kinetics across BBB from apical to basolateral and from basolateral to aoical, respectively; and *Cl*_*efflux*_, *Cl*_*metabolism*_ and *Q*_*convection*_ represent the individual clearance mechanisms indicated.

1 $${}^{CSF}C_{{}} = \frac{{R_{in} }}{{CL_{out} }} = \frac{{PS_{A\;to\;B} \times {}^{serum}C_{{}} }}{{PS_{B\;to\;A} + CL_{Efflux} + CL_{metabolism} + Q_{convection} }}$$

Without consumption of energy, passive diffusion or facilitated transcytosis requires a favorable concentration gradient and occurs only from high to low concentrations. Therefore, passive diffusion out of the CNS is not a feasible clearance mechanism for antibodies. Thus far, there has been no reported evidence for a high efficiency efflux pump or massive brain specific catabolism for antibodies or human IgGs. Unlike the other clearance mechanisms described above, convection by ISF/CSF flow does not require a favorable concentration gradient, specific transporter, or energy and is not structurally restricted by stoichiometry. If one assumes that convection is the most plausible and efficient mechanism for CNS removal of antibodies, Eq. 1 can be re-arranged and simplified to Eq. 2.

2 $$\frac{{{}^{CSF}C_{ss} }}{{{}^{serum}C_{ss} }} \approx \frac{{PS_{A\;to\;B} }}{{Q_{convection} }} \approx 0.1\%$$

In this model, the major factors that determine CNS exposures of therapeutic antibodies are two independent processes: an antibody’s ability to cross the BBB, and clearance dominated by the mechanism of convection, which is closely associated with the brain’s ISF/CSF production rate. Assuming rapid equilibrium across the various CNS compartments and CSF antibody concentration as a surrogate for CNS drug levels, the pharmacokinetic modeling of CNS antibody concentration can be simplified to Eq. 3, where ^*CNS*^*V* is the apparent volume of distribution for CNS, ^*CSF*^*C* and ^*serum*^*C* denote the antibody concentrations in CSF and serum, respectively, and *PS*_*product*_ and *Q*_*convection*_ are independent parameters that govern entering and exiting the CNS. Entering CNS (*PS*_*product*_) requires overcoming the BBB/BCSFB, whereas exiting CNS (*Q*_*convection*_) to a large extent does not. Both processes are independent of systemic clearance mechanisms.

3 $$\frac{{{}^{CNS}V_{{}} d{}^{CSF}C}}{dt} = PS_{product} \times {}^{serum}C_{{}} - Q_{convection} \times {}^{CSF}C_{{}}$$

The apparent linearity of the predicted vs. experimentally measured serum and CSF data for antibody A in Fig. 3b, c confirm that the model has little bias over a wide range of antibody concentrations. Although the variation in serum pharmacokinetic profiles are small (Fig. 3b), the differences in CSF to serum ratios are relatively large (Fig. 3c). Similar variations were observed for antibody C (Fig. 4a). For the 12 monkeys that received 30 mg/kg of antibody C, ^*CSF*^*AUC*-*to*-^*serum*^*AUC* ratios ranged from 0.03 to 0.23%.

## Discussion

The CSF-to-serum ratio of 0.1–0.2% that we measured for five independent antibodies in rats and NHPs agrees well with published results for other antibodies. CSF-to-total serum IgG ratios of 0.1–0.2% in healthy humans have been reported by several groups [11–13]. Similarly, for therapeutic antibodies in humans, there are many reports from sparse CSF sampling that was used to estimate CSF-to-serum ratios. For example, in patients treated with rituximab for neurological autoimmune diseases, the (lumbar) CSF to serum antibody ratios were approximately 0.1–0.2% [14]. Trastuzumab had a CSF-to-serum ratio of 0.3% in a 62-year-old patient with meningeal carcinomatosis treated by weekly IV administration [15]. MGAWN1, an antibody against West Nile virus, had a CSF-to-serum ratio of 0.25% in healthy volunteers [16]. CSF to serum ratios for anti-LINGO-1 mAb BIIB033 measured 2 weeks post dose are 0.032–0.079% in healthy volunteers and 0.03–0.13% in individuals with multiple sclerosis [17]. Finally, a murine analog of anti-amyloid beta mAb solanezumab had a CSF to plasma ratio in rats of approximately 0.1% [18]. Of special note, the CSF to serum ratios of 0.032–0.079% for BIIB033 (antibody E) observed in healthy volunteers, matches well with the 0.075% value we report here in rats for the same antibody.

There are many studies exploring the mechanisms by which antibodies cross the BBB [19]. Garg and Balthasar studied antibody distribution in neonatal Fc receptor (FcRn) deficient mice. Although the plasma AUC of ^125^I-labeled antibody was significantly reduced in FcRn−/− mice, the brain-to-plasma exposures remained unchanged at 0.22% in wildtype and FcRn−/− mice. Similar to what we observed in rats and NHP, the brain concentration–time profiles paralleled the plasma profiles in wildtype and FcRn−/− mice (i.e., ^*brain*^*t*_*½*_ was equal to ^*plasma*^*t*_*½*_) [20]. Other proposed mechanisms include a low-rate nonspecific adsorptive mechanism involving transcytosis, endocytosis, or macropinocytosis [20–23]. Unlike with small molecules, it is challenging to accurately measure antibody concentrations in brain tissues. With CNS-to-serum concentration ratios for antibodies of only 0.1–0.2%, it is critical to prepare brain tissues that are free of residual blood. Brain ISF levels can be determined by microdialysis, but this is a technically challenging method for antibodies due to their large size. Thus, brain tissue data provides a rough estimate of CNS drug levels but must be interpreted with caution. In both preclinical and clinical settings, CSF has been widely accepted as a surrogate for assessing CNS drug levels [2, 5, 24]. In investigating CNS exposure levels in animals, euthanasia followed by perfusion and collection of brain and CSF samples could impact the data. Further work is needed to understand potential differences that might arise due to sample handling.

**Fig. 6:**
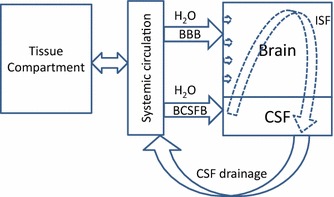
Diagram for water flow into and out of the CNS

Although CSF is largely produced at the choroid plexus (CP) and secreted into the four ventricles of the brain, recent studies indicate there are other sources of CSF [24–27]. As illustrated in Fig. 6, water is secreted into the CNS by endothelial cells of the BBB, forming brain ISF, and by choroid plexus (CP) into ventricles, forming CSF [25, 28]. CSF also enters the brain interstitial spaces via peri-capillary (Virchow–Robin) spaces [27, 28]. The junctures between brain parenchyma and CSF interfaces are relatively loose and allow exchange of solutes between brain ISF and CSF [24, 29, 30]. Moving away from BBB and BCSFB, ISF/CSF carries solutes and eventually drains into blood at multiple locations, including the dural sinus [27, 31, 32]. There has been no report that BBB and/or BCSFB are involved in CSF drainage/reabsorption.

For the factors that determine the CNS exposure of an antibody, *PS*_*product*_ and *Q*_*convection*_ are two independent processes that cannot be simplified by inter-compartmental clearance and are independent of systemic clearance mechanisms. Large inter-subject variations in CNS antibody exposures need to be taken into consideration even if there is little variation in a drug’s serum concentration–time profile. Variation can be introduced at the CNS level by an individual’s *PS*_*product*_*/Q*_*convection*_ ratio. From a pharmacokinetic modeling point of view, these two parameters are highly correlated and difficult to differentiate other than through a change in their ratio. That said, the *PS*_*product*_ value of an antibody can be assessed using in vitro methods [23] and an individual’s CSF production rate can be assessed by MRI [33]. On average, rats have 150 µL CSF by volume, which undergoes turnover 11 times per day. In contrast, healthy humans have an average CSF volume of 580 mL and a turnover rate of 4 times per day [1]. The CSF turnover rate sets the efficiency of convective clearance out of the CNS. It is well documented that CSF production rates vary under different conditions, including circadian rhythm [33–36], normal aging [36–39], concurrent medications such as the carbonic anhydrase inhibitor acetazolamide [40, 41] and the proton pump inhibitor omeprazole [42, 43], and in disease conditions such as hydrocephalus [26, 44] and Alzheimer’s disease (AD) [45, 46]. Alterations in CSF production rates can have profound effects on CNS clearance of medications and waste products. In individuals with AD, reduced brain ISF/CSF turnover is probably caused by decreased brain ISF/CSF production and increased CSF volume [45, 46]. BAN2401, a monoclonal antibody targeting amyloid beta protein, has a CSF-to-serum ratio in AD patients of 0.04–0.08% at 24 h following dose administration, but increases to 0.29–0.81% after 10–14 days [47]. These data suggest that ^*CSF*^*T*_*max*_ for BAN2401 is > 24 h and that ^*CSF*^*C* levels have not yet peaked 24 h after dose administration. CSF-to-serum ratios of greater than 0.1–0.2% have also been reported for other antibody therapies in individuals with AD [13, 48, 49]. CSF-to-serum total albumin and IgG ratios are also elevated in AD patients [12, 13], consistent with AD patients has reduced CSF clearance rates [45, 46].

Linking CSF production rates with CNS clearance of antibodies potentially has broad applications, since it enables the estimation of antibody kinetics between the CSF and systemic circulation and would allow for rational design of CSF sampling schedules and projection of CNS drug exposures in selected patient groups. For example, for antibodies, delayed ^*CSF*^*T*_*max*_ and steady-state CSF-to-serum concentration ratios are observed. These should be taken into consideration when designing CSF sampling schemes. In patients with reduced CSF production, either due to disease or concurrent medications, higher CSF drug exposures should be expected. Unfortunately, the factors that affect CSF production rates and, more importantly, the subsequent impact on their ability to clear CNS waste have not yet been fully investigated and/or appreciated.

In summary, from an extensive analysis of the pharmacokinetics of five human antibodies in rats and monkeys, we show that antibodies enter the CNS slowly, with ^*CSF*^*T*_*max*_ around 24–72 h after IV administration, and that CSF and serum concentration–time curves become parallel after ^*CSF*^*T*_*max*_, with average CSF-to-serum ratios of 0.1–0.2%. Prior to achieving ^*CSF*^*T*_*max*_, CSF-to-serum ratios are not accurate indicators of CNS uptake or steady-state CSF-to-systemic concentration ratios. For antibodies with long systemic *t*_*½*_s, ^*serum*^*t*_*½*_ determines ^*CSF*^*t*_*½*_. These studies should aid in the design of future studies to better understand factors that contribute to the elimination of antibodies from the CNS.

## Abbreviations

BBB: blood–brain-barrier
CSF: cerebrospinal fluid
ISF: interstitial fluid
BCSFB: blood–CSF-barrier
mAb: monoclonal antibody
